# Application of Coating Chitosan Derivatives (N,O–Carboxymethyl Chitosan/Chitosan Oligomer Saccharide) in Combination with Polyvinyl Alcohol Solutions to Preserve Fresh Ngoc Linh Ginseng Quality

**DOI:** 10.3390/foods12214012

**Published:** 2023-11-02

**Authors:** Ngoc Nguyen, Trieu Nguyen, Phu Le Hong, Thi Kieu Hanh Ta, Bach Thang Phan, Hanh Nguyen Thi Ngoc, Hang Phung Thi Bich, Nhi Dinh Yen, Toi Vo Van, Hiep Thi Nguyen, Diep Tran Thi Ngoc

**Affiliations:** 1 School of Biomedical Engineering, International University, Ho Chi Minh 700000, Vietnam; nttngoc@hcmiu.edu.vn (N.N.);; 2 Vietnam National University, Ho Chi Minh City 700000, Vietnam; 3 Shared Research Facilities, West Virginia University, Morgantown, WV 26506, USA; 4 School of Biotechnology, International University, Ho Chi Minh 700000, Vietnam; 5 Faculty of Materials Science and Technology, University of Science, Ho Chi Minh City 700000, Vietnam; 6 Center for Innovative Materials and Architectures, Ho Chi Minh 700000, Vietnam; 7 Centre for Innovation and Technology Transfer, International University, Ho Chi Minh 700000, Vietnam

**Keywords:** Ngoc Linh ginseng (NL ginseng), Chitosan (CS), N,O–carboxymethyl chitosan (NOCC), chitosan oligomer saccharide (COS), Polyvinyl alcohol (PVA), coating, preservation

## Abstract

The postharvest preservation of Ngoc Linh ginseng (NL ginseng) is essential to retain its quality and sensory values for prolonged storage. In this study, the efficacy of NL ginseng preservation by coating chitosan derivatives in combination with polyvinyl alcohol (PVA) solutions was investigated under refrigeration conditions (~3 °C; ~40% RH) for 56 days. The effect of the chitosan–based solutions, including N,O–carboxymethyl chitosan (NOCC), chitosan oligomer saccharide (COS), or chitosan (CS), and the blend solutions (NOCC–PVA or COS–PVA) on the coated NL ginsengs was observed during storage. The pH values, viscosity, and film-forming capability of the coating solutions were determined, while the visual appearance, morphology, and mechanical properties of the films formed on glass substrates as a ginseng model for coating were also observed. The appearance, skin lightness, weight loss, sensory evaluation, total saponin content (TSC), total polyphenol content (TPC), and total antioxidant capacity (TAC) of the coated NL ginsengs were evaluated. The findings showed that the observed values of the coated NL ginsengs were better than those of the non-coated samples, with the exception of the COS–coated samples, which had completely negative results. Furthermore, the NOCC–PVA solution exhibited a better preservation effect compared with the COS–PVA one based on the observed indices, except for TPC and TAC, which were not impacted by the coating. Notably, the optimal preservation time was determined to be 35 days. This study presents promising preservation technology using the coating solution of NOCC–PVA, harnessing the synergistic effect of pH 7.4 and the form–firming capacity, to maintain the shelf life, medicinal content, and sensory attributes of NL ginseng.

## 1. Introduction

Ngoc Linh ginseng (*Panax vietnamensis* Ha et Grushv.) is a precious medicinal herb of Vietnam [1,2,3]. NL ginseng has been renowned for its composition of 52 ginsenosides or saponins, of which ocotillo-type ginsenosides are the most abundant [4]. For traditional medicine, similar to other types of ginseng, NL ginseng has been reserved as a versatile remedy for various ailments [5,6]. Recently, various biological activities from NL ginseng and its compounds such as anti-melanogenic [7], antitumor [8,9], hepatoprotective [10,11], and pain and stress relief were reported [12]. However, fresh NL ginseng is susceptible to experiencing fresh weight loss, rapid spoilage, and phytochemical degradation, which ultimately leads to a decline in both its quality and market value [13,14]. Hence, there is an urgent demand to develop preservation techniques to maintain their values and meet consumer expectations. As expected, the existing literature lacks reports on preservation technology specifically tailored for fresh NL ginseng.

In recent decades, the food preservation industry has become increasingly interested in the use of chitosan, a substance derived from crustaceans such as shrimps or crabs [15]. Chitosan has been successfully applied as a technical coating for preserving various fruits, such as apples [16], tomatoes [17], and cherries [18], owning to its antibacterial activity [19], barrier properties [20], non–toxicity [21], and eco–friendliness [21,22]. However, chitosan’s applications could be restricted due to its high molecular weight and the presence of amino groups, resulting in poor solubility at a neutral pH and weak homogeneity with other substances [23,24]. To address the drawbacks of chitosan, chitosan derivatives, such as N, O–carboxymethyl chitosan (NOCC) and chitosan oligomer saccharide (COS), have been developed by modifying the chemical structure of chitosan for broader applications [25]. NOCC, created by introducing carboxymethyl groups into the hydroxyl and amino groups of a chitosan polymer, exhibits enhanced solubility in acidic, neutral, and alkaline conditions [26]. It was also known for its antimicrobial activities against a wide range of bacteria, low toxicity, and high biocompatibility, making it a potential material for fruit preservation [27,28]. A recent study showed that tomatoes coated with (2% *w*/*v*) chitosan or NOCC solutions maintained their physical and chemical characteristics, delayed the ripening process, and prolonged storage time [22]. Similarly, COS, created through the enzymatic or chemical hydrolysis of chitosan to acquire a lower molecular weight, is easily water-soluble, non–toxic, and antibacterial [29,30]. In another study, strawberry fruits treated with a COS solution (100 mg/L) maintained a prolonged shelf life and their overall quality during the storage period [31]. Nevertheless, similar to other natural polymers, the use of chitosan–based coating solutions presents several drawbacks, such as inadequate mechanical barrier properties, high water vapor permeability, high rigidity, and so forth [32].

On the other hand, numerous studies have demonstrated that the addition of synthetic polymers to natural polymers for coating techniques offers attractive alternatives [33,34]. One such example is polyvinyl alcohol (PVA), which is a synthetic and water-soluble polymer known for its excellent biodegradability, non-toxicity, and film-forming properties [35]. PVA is also considered an eco-friendly packaging material due to its favorable physiochemical and elastic characteristics [36]. It was noted that PVA can be tailored with chitosan derivatives to achieve optimal characteristics for coating techniques. For instance, one recent study reported that a mixture of carboxymethyl chitosan (CMCS) and PVA was applied as an edible coating to effectively extend the shelf life and freshness of orange fruits [37]. Moreover, membranes synthesized from CMSC/PVA materials possessed high tensile strength, making them a promising packaging material for fruit preservation. In other findings, blended solutions comprised of PVA and chitosan derivatives exhibited excellent viscosity, film formation, biodegradability, and antibacterial properties [38,39]. Given the extensive research on these methods in various fruit products, the utilization of these mixed solutions to preserve fresh ginsengs, including NL ginseng, has yet to be investigated.

We present, to the best of our knowledge, a novel study on a coating method for preserving NL ginseng under cold storage conditions (~3 °C; ~40% RH). Our goal is to preserve the quality and sensory values of NL ginseng for long-term storage. The main works are mainly twofold: (1) to assess the effect of chitosan-based solutions (NOCC, COS, or CS) and blend solutions (NOCC–PVA or COS–PVA) on the appearance of NL ginseng after 35 days of storage, and (2) to further investigate the effect of these blend solutions on its skin lightness, weight loss, sensory evaluation, and phytochemical content properties during storage for up to 56 days.

## 2. Materials and Methods

### 2.1. Materials

Ngoc Linh ginseng, comprising roots and rhizomes and cultivated for ten years, was supplied by Dak To Forestry Co., Ltd. (Kon Tum, Vietnam).

All chemicals used were analytical and supplied from commercial sources as follows: Chitosan (CS, shrimp shells; ≥deacetylated) and Polyvinyl alcohol (PVA, Mw 89,000–99,000, 99+% hydrolyzed) from Sigma Aldrich (Waltham, MO, USA). Isopropanol alcohol (IPA), sodium hydroxide (NaOH), acid hydrochloric (HCl), and acetic acid (CH3COOH) from Xilong Chemical Co., Ltd. (Shantou, China). Chloroacetic acid (ClCH2COOH) from HiMedia Laboratories Pvt., Ltd. (Thane, India). In addition, chitosan oligo saccharide (COS, Chitosan CTIC15; 15.000–18.000 ppm) was supplied from Vietnam Food JSC (Ho Chi Minh, Vietnam). Distilled water was obtained from the International University—Vietnam National University in Ho Chi Minh city (Vietnam).

Molecular porous membrane tubing (Spectra/Por^®^ 4 dialysis membrane, MWCO:12–14 kDa) was obtained from Spectrum Laboratories, Inc. (Rancho Dominguez, CA, USA). Microscope Slides were purchased from Waldemar Knittel Co. (Braunschweig, Germany). Double-ring filter paper was purchased from Cytiva Co. (Guangzhou, China). PP plastic boxes (HT-17, 22 × 14 × 6 cm) were purchased from Hunufa Co., Ltd. (Ho Chi Minh, Vietnam).

### 2.2. Coating Solution Preparation

The synthesis of NOCC was successfully conducted according to established protocols with minor adjustments [40,41]. Generally, NOCC was created by adding carboxymethyl groups to the N– and O–positions of the glucosamine and N–acetylglucosamine units of CS to a substitution degree of 85%. Firstly, 2 g of CS was soaked in 10 mL IPA solution at room temperature for 1 min. Then, 10 mL of 13N NaOH solution was poured into the CS mixture for the 1-h rest. Simultaneously, the CAA solution was prepared by dissolving 5.2 g of CAA powder in the 10 mL IPA solution. The CS mixture was then heated in a water bath at 600 °C and left to react for 3 h. After that, 2 mL of 52% *w/v* CAA solution was added to the mixture every 5 min for five times, and the solution was filtered to collect the residual solid product. The solid product was redissolved in distilled water and neutralized by 2.5 M HCl solution. This solution was dialyzed for two days and lyophilized to obtain the final NOCC product.

PVA solutions (6% *w*/*v*, 12% *w*/*v*, and 6.6% *w*/*v*) were prepared by dissolving PVA powder in distilled water and stirring at 400 rpm at 90 °C for three hours. Simultaneously, NOCC solutions (4% *w*/*v*, 2% *w*/*v*, and 1% *w*/*v*) were achieved by dissolving NOCC powder in distilled water and stirring at 400 rpm for 1 day at room temperature. The NOCC1–PVA6 solution was then achieved by mixing NOCC 2% *w/v* and PVA 12% *w/v* at 1:1 ratio. Meanwhile, COS solution (1% *v*/*v*) was obtained by diluting the stock COS solution 10 times with distilled water, and the COS1–PVA6 solution was also prepared by mixing 5 mL of the stock COS solution with 45 mL of 6.6% *w/v* PVA solution. CS2 solution (2% *w*/*v*) was achieved by dissolving CS powder in acetic acid (1% *v*/*v*), stirring for 4 h, and adjusting to pH 5.6 by 1 M NaOH solution. All sample formulations are detailed in Table 1.

### 2.3. Film Preparation

The dip–coating method was built upon previous research, with some minor modifications [42]. Microscope slides, used as glass substrates, were dipped into the coating solutions for 1 min and then placed in a vacuum oven at 37 °C. An exception was made for the NOCC4 coating technique, which required the use of a casting tool to spread the suspension on the surface evenly. After 24 h, thin films were formed and carefully removed from the glass substrate’s surface for subsequent characterizations.

### 2.4. Coating Processing and Storing Application of NL Ginseng

**Figure 1:**
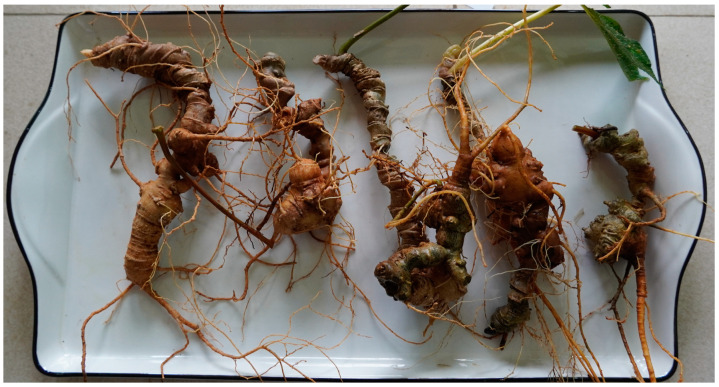
The fresh NL ginsengs before storage.

Fresh NL ginsengs were packed into cotton boxes covered with moisture paper tissue and ice gel, which is equivalent to cold conditions (~12 °C, ~50% RH) during transporting from Kon Tum province to the laboratory. Figure 1 shows fresh NL ginsengs after completing the preparation process.

The coating and storing procedures of NL ginseng followed previous protocols, with some modifications [43,44]. NL ginsengs were washed to remove any remnant soil and dust. They were then soaked in a NaCl solution (2% *w*/*v*) for 5 min, followed by rinsing with distilled water and drying with soft cotton linen paper. To ensure complete drying, these ginsengs were placed in a refrigerator (Aqua, 90 Lit-AQR-D99FA, Aqua Co., Ltd., Biên Hòa, Vietnam) for 5 h. Next, they were immersed in the coating solutions for 1 min and subsequently air-dried at ambient temperature. Non-coated NL ginsengs were used as control groups. All NL ginsengs were placed in plastic boxes, whose lids were punched with 10 holes (diameter 100 µm) and stored in a refrigerator set (~3 °C, ~40% RH) for 35 to 56 days. The refrigerator’s condition was monitored by a temperature and humidity data logger (Elitech-Version 6.2.0, Elitech, Logan, UT, USA).

### 2.5. Characterizations

#### 2.5.1. Viscosity

The viscosity of the coating solutions was measured by Viscometer (Brookfield RVDVI+, Middleboro, MA, USA). In each test, 11 mL of sample was used with no. 28 hanging spindle and rotating speed at 100 rpm.

#### 2.5.2. pH

The pH value of the coating solutions was determined by a pH meter (Laqua PH210, Horiba, Osaka, Japan).

#### 2.5.3. Fourier-Transform Infrared Spectroscopy

The functional groups in the coating solutions were determined by FTIR spectra (Bruker Vertex 70v FT-IR, Billerica, MA, USA) within the wavenumber range of 4000–400 cm^−1^.

#### 2.5.4. Tensile Strength

The mechanical property of the films was tested using a Texture Analyzer (Stable Micro Systems v.TA-XT plus, London, UK) where 5 × 1 cm rectangular films were held vertically leaving 2 cm distance between two grips. The film thickness was determined by Scanning Electron Microscope (SEM). The tests were performed with the crosshead speed at 5 mm/s.

#### 2.5.5. Morphology

The surface morphology of the films was observed by an optical microscope (Eclipse Ti-U series, Nikon, Tokyo, Japan) at 4× magnification.

The cross-section morphology of the films and NL ginseng’s skin and fresh were observed by SEM (JSM-IT100, JEOL, Tokyo, Japan) at 10 kV accelerating voltage. Additionally, samples were fixed with liquid nitrogen before sputter coated with gold (Smart Coater, JEOL, Tokyo, Japan).

NL ginsengs were also captured by a photographic camera (Samsung Note 22 Ultra, Samsung, Gyeonggi, Republic of Korea) in natural lighting conditions.

#### 2.5.6. Weight Loss

The weight loss of NL ginsengs was followed previous research with minor modifications [45]. The weight loss of NL ginsengs was determined after 7, 35, and 56 storage days using an electronic balance (BL-320S, Shimadzu, Kawasaki, Japan). The weight loss percentage was calculated as:

$$Weight\;loss\;\left(\%\right)=\frac{\left(inital\;weight-observed\;weight\right)}{initial\;weight}\times 100$$

#### 2.5.7. Skin Lightness

The skin lightness of NL ginsengs was evaluated using a colorimeter (JZ-300 Universal Color Meter, Shenzhen King Well Instrument, Guangdong, China), referencing from established research [14,46]. Chromaticity values on three different positions were determined. Calibration was conducted with a white tile and a black light trap tile before sample measurement. L* values (Lightness: 0 = black, 100 = white) were recorded.

#### 2.5.8. Sensory Evaluation

The sensory evaluation of NL ginsengs was carried out according to the previous protocol with minor modifications [47]. Sensory evaluation attributes, including texture, color, flavor, and overall acceptability, were judged by three experts on a 9-point hedonic scale. The sensory preference ranges from 1–2 Poor, 3–4 Fair, 5–6 Good, 7–8 Very good, and 9 Excellent. The experiment was conducted in the evaluation sensory room (~25 °C, ~60% RH).

#### 2.5.9. The Total Phytochemical Components

NL ginseng was extracted according to previous processing with some adjustments [3,48]. NL ginseng samples were vacuum–dried at 60 °C until reaching a constant weight. Subsequently, 1 g of dried NL ginseng was immersed in 30 mL ethanol (75% *v*/*v*) and shaken at 250 rpm, 80 °C for 2 h. Following this, the mixture was centrifuged to obtain the extract, and the solid residue continued to be extracted with 10 mL ethanol (75% *v*/*v*) with the same parameters. The second extraction process was repeated 7 times. Finally, all the resulting extracts were mixed together as the final product.

The total saponin content (TSC) was determined by the vanillin–sulfuric acid assay according to previous research with minor modifications [49]. Firstly, 0.5 mL of 8–fold diluted sample extract was mixed with 0.5 mL of 8% *w/v* vanillin solution, prepared by dissolving powder in 99.9% methanol. The mixture was put in cold water bath and gradually added with 5 mL of H2SO4 solution (72% *v*/*v*), then temperature was raised to 75 °C to catalyze the reaction in 15 min. Finally, the solution was cooled to room temperature and the absorbance was measured at 544 nm using a UV-Vis spectrophotometer (UVD-3500, Labomed, Los Angeles, CA, USA). Oleanoic acid was used as a standard. TSC was expressed as mg olenoic acid equivalent/g of dry matter (mg OAE/gdw) and calculated by the following formula: y = 3.8134x + 0.0169.

The total antioxidant capacity (TAC) was determined by DPPH (2,2-diphenyl-1-picrylhydrazyl-hydrate) radical scavenging assay to estimate the radical scavenging activity of NL ginseng extract according to the previous method with minor modifications [48]. DPPH solution (0.15 mM) was prepared by being completely dissolved in ethanol (96%). Next, 2 mL of the 20 mg/mL ginseng extract was diluted by 0, 1, 2, 4, and 8 times. Then, 2 mL of the DPPH solution was mixed with 2 mL of the diluted extracts, and the mixtures were kept for 30 min at room temperature in the dark. The absorbance was measured at 517 nm by UV-Vis spectrophotometer, using 2 mL ethanol (96%) as the blank. Ascorbic acid was used as standard. TAC was expressed as mg ascorbic acid equivalent/g of dry matter (mg AAE/gdw) and calculated by the following formula: y = 49.7x + 0.0073.

The total polyphenol content (TPC) was determined by the Folin–Ciocalteau method according to previous research with minor modifications [50]. Firstly, 0.5 mL of extract was dissolved in 2.5 mL of the Folin–Denis solution (10% *v*/*v*) for 5 min. The mixture was then added with 2 mL of the Na_2_CO_3_ solution (7.5% *w*/*v*) for 60 min in the dark. Absorbance was measured at 760 nm with a UV-Vis spectrophotometer. Gallic acid was used as a standard. TPC was expressed as milligrams of gallic acid equivalents/g of dry matter (mg GAE/gdw) and calculated using the formula: y = 19.43x − 0.0687.

#### 2.5.10. Statistical Analysis

Data were presented as mean ± standard deviation (SD). The statistical analysis of the obtained data was performed using the t–test and the one–way ANOVA method. Difference between groups was considered statistically significant when *p* < 0.05. All the tests were repeated to achieve statistical reliability unless stated otherwise.

## 3. Results

### 3.1. Analysis of Coating Solutions

#### 3.1.1. Chemical Functional Groups

**Figure 2:**
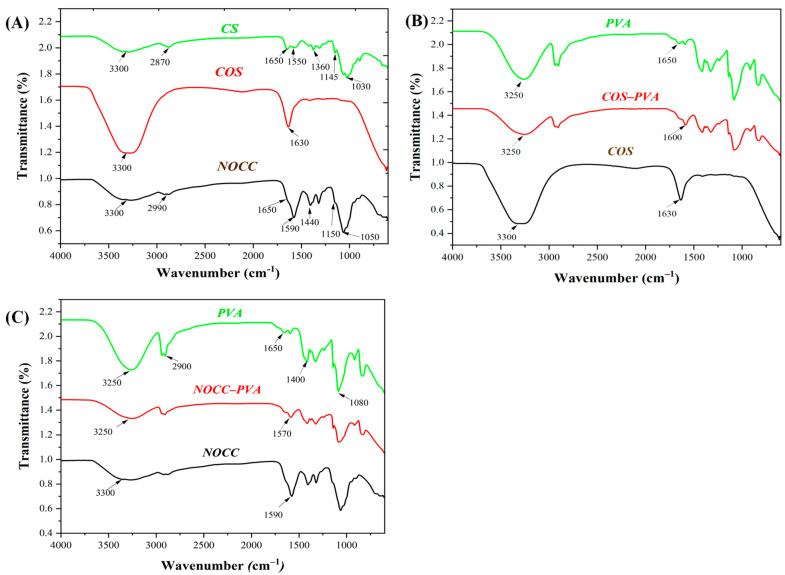
FTIR spectra of the coating solutions: (**A**) The chitosan-based solutions: CS, NOCC, and COS. (**B**) Comparison of the NOCC–PVA solution with the PVA and NOCC solutions. (**C**) Comparison of the COS–PVA solution with the PVA and COS solutions.

FTIR spectra of the chitosan-based solutions are analyzed in Figure 2A to determine their functional groups. The CS solution exhibited several characteristic peaks around 3300 cm^−1^, which corresponded to the N–H stretching vibration of the amino groups (–NH2) and the –OH stretching vibration of hydroxyl groups; 1650 cm^−1^, representing the C=O stretching vibration of the acetyl groups (COCH3); 1550 cm^−1^ due to the bending vibration of the NH2 groups; 1360 cm^−1^, indicating the C–H bending vibration of the CH2 groups; 1145 cm^−1^ associated with the C–O stretching vibration of the glycosidic bonds; and 1030 cm^–1^, attributed to the C–N stretching vibration of the amine groups [16]. In the NOCC solution, apart from the chitosan peaks in NOCC, there was a replacement of peaks around (1650 cm^−1^ and 1550 cm^−1^) to 1590 cm^−1^, and 1030 cm^−1^ to 1150 cm^−1^, indicating the presence of carboxymethyl groups [26]. On the other hand, the COS solution only had two major peaks at 3300 cm^−1^, due to the N–H stretching vibration of the amino groups (–NH2) and the –OH stretching vibration of hydroxyl groups, and 1630 cm^−1^, related to the C=O stretching vibration of the acetyl groups. Notably, these peaks were of a higher intensity than those of chitosan.

Furthermore, the FTIR spectra of the blend solutions (COS–PVA and NOCC–PVA) were compared to those of their individual components, illustrating the interactions between their chemical functional groups in Figure 2B,C. In addition, the PVA solution had peaks at around 3250 cm^−1^ due to the stretching vibration of the –OH groups; 2900 cm^−1^, representing the C–H stretching vibration of the CH2 groups; 1650 cm^−1^, associated with the stretching vibration of the carbonyl groups (C=O); and 1400 cm^−1^, corresponding to the bending vibration of the CH2 groups [51,52]. Compared to the PVA solution and only chitosan-derived solutions, both the NOCC–PVA and COS–PVA solutions showed a significant decrease in intensity at peaks of around 3250 cm^–1^ and a slight reduction in wavenumber to 1570 cm^−1^ and 1600 cm^−1^, respectively. These changes suggest interactions between the components within the blend solutions [53,54].

#### 3.1.2. pH

The coating solutions’ pH values are depicted in Table 1. These pH values were primarily determined by the kind of chitosan materials used. In detail, the chitosan and its derivatives solution exhibited different pH values, with CS2 (pH 5.6), COS (pH 4.2), and both NOCC1 and NOCC4 (pH 7.4). As for the blend solutions, the pH values of NOCC1–PVA6 and COS1–PVA6 were 7.4 and 4.6, respectively. Additionally, the PVA solution itself had a pH of 6.5, which acted as a moderate acid. Notably, when combined with COS, the PVA solution slightly reduced the acidity of the COS solution, while there was no impact on the neutral pH of the NOCC solution.

#### 3.1.3. Viscosity and Film-Forming Capability

Viscosity values of all coating solutions are shown in Table 1. Additionally, this analysis also evaluated their film-forming capability on glass substrates through visual observation. In the case of solutions containing only chitosan, namely CS2, NOCC1, and COS1, their low viscosities were not measurable under established parameters, and presented the absence of a film–forming property.

In contrast, the remaining four solutions (NOCC4, NOCC1–PVA6, COS1–PVA6, and PVA6) were capable of forming good films due to their enhanced viscosity values. Among them, the NOCC4 solution reached the highest viscosity of 1700 cP, allowing the formation of a thin film under the support of a casting tool to spread suspension evenly throughout the substrate. Despite having the lowest viscosity of 25 cP, the PVA6 solution still managed to create a thin and smooth film.

In the case of the mixed solutions, the addition of PVA significantly enhanced the viscosity of the NOCC or COS solutions. Consequently, both the NOCC1–PVA6 and COS1–PVA6 solutions exhibited comparable viscosities of 240 cP and 185 cP, respectively.

### 3.2. Analysis of Film’s Characteristics

#### 3.2.1. Morphology Property

**Figure 3:**
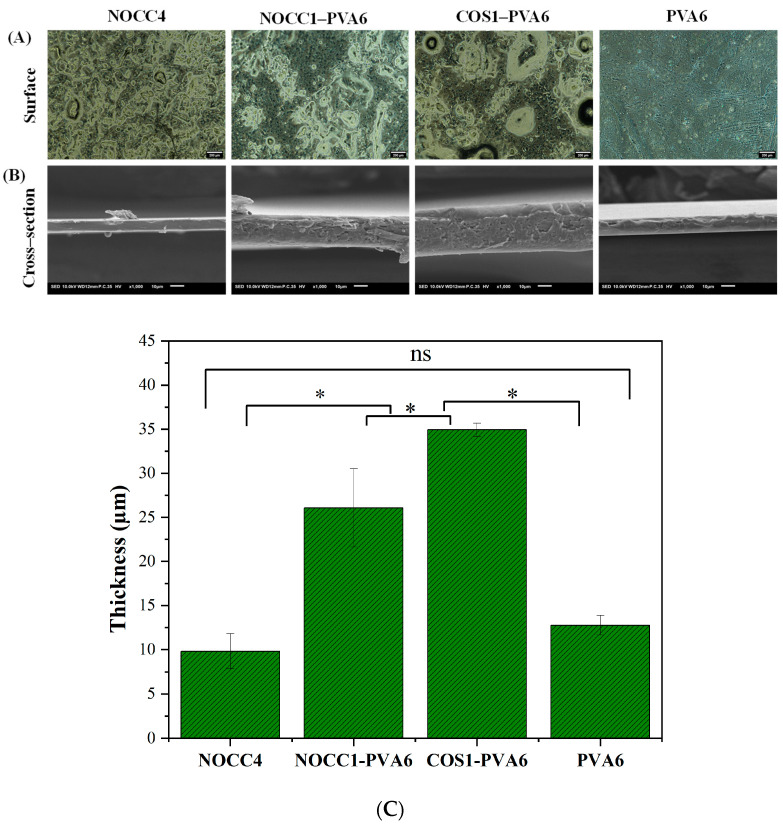
SEM morphology of the NOCC4, NOCC1–PVA6, COS1–PVA6, and PVA6 films: (**A**) Optical microscope image of the films’ surface (scale bar 200 µm). (**B**) SEM image of the films’ cross–section (scale bar 20 µm). (**C**) Thickness of the films’ cross–section. The data present as Mean ± SD (*n* = 3, *: *p* < 0.05, ns: *p* > 0.05).

Optical microscope images of the films (NOCC4, NOCC1–PVA6, COS1–PVA6, and PVA6) are shown in Figure 3A. This analysis provided insights into the surface morphology of the films. The NOCC4 image exhibited the uniform distribution of agglomerated particles, which was also known as a characteristic of the crystalline structure [55]. In contrast, the PVA6 image revealed two slightly distinguishable areas: large smooth areas and small agglomeration areas, representing the semi-crystalline structure [56,57]. As for the blend films, both NOCC1–PVA6 and COS1–PVA6 exhibited the presence of two distinct phases, as mentioned above, indicating the semi–crystalline structure with a high density of agglomeration. Notably, the mixing process induced the formation of voids within the films.

SEM images of these films’ cross–section are also shown in Figure 3B. The NOCC4 and PVA6 films both exhibited solid and homogenous morphologies, with the NOCC4 film having a flat topography and the PVA film showing some unevenness. On the other hand, the composite films displayed porous structures with their rough and uneven terrains.

Additionally, their thickness is depicted in Figure 3C. The thickness values of the NOCC1–PVA6 and COS1–PVA6 films (*p* < 0.05) were 26±4.4 µm and 34.9±0.7 µm, respectively, which were significantly higher than the PVA6 film at 12.7±1.1 µm. Finally, the thickness of the NOCC4 film was 9.8±1.9 µm; however, this discrepancy should not be a cause of concern as the different coating techniques used.

#### 3.2.2. Mechanical Property

**Figure 4:**
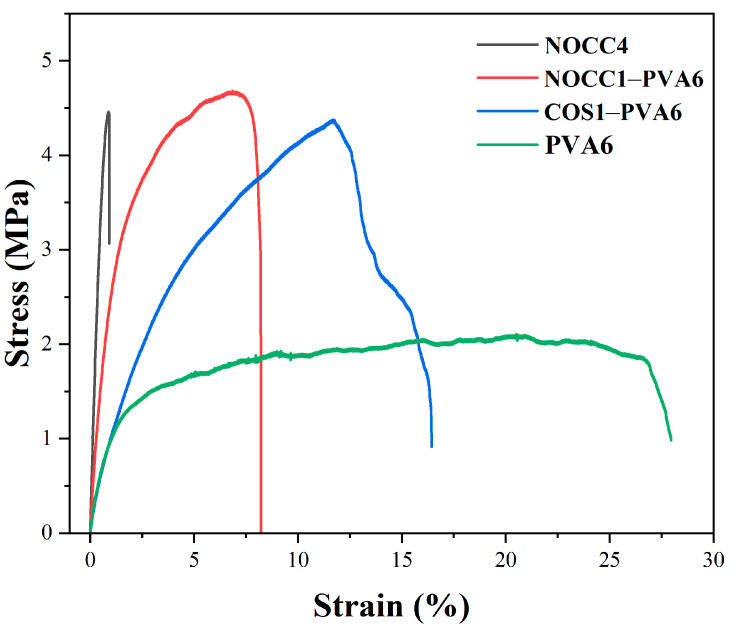
Stress–strain curves of the NOCC4, NOCC1–PVA6, COS1–PVA6, and PVA6 films. The graph represents one of three resets.

Stress–strain curves of the produced films are shown in Figure 4, and the corresponding stress (MPa) and elongation (%) at a break are presented in Table 2. The mechanical behavior of the NOCC4 film was relatively brittle, exhibiting a very low elongation at a break of 0.87% and a high stress value at a break of 4.46 MPa. On the contrary, the PVA6 film was more stretchable and resistant, evidenced by high elongation at a break of 20.51%, although it had a lower stress value at a break of 2.11 MPa.

Remarkably, the blend films exhibited the combination of mechanical advantages from both their constituents. The NOCC1–PVA6 film demonstrated a significant improvement on mechanical behavior, with the stress and elongation values at the break reaching 4.68 MPa and 6.84%, respectively. Similarly, the COS1–PVA6 film also achieved comparable stress at a break of 4.38 MPa, but higher elongation at a break measuring up to 11.72%.

### 3.3. Preservation Results of NL Ginseng

#### 3.3.1. Appearance of NL Ginseng after Preservation

After the completion of 35–day preservation, SEM images were taken to examine the effects of different coatings on both the skin and flesh of NL ginsengs.

**Figure 5:**
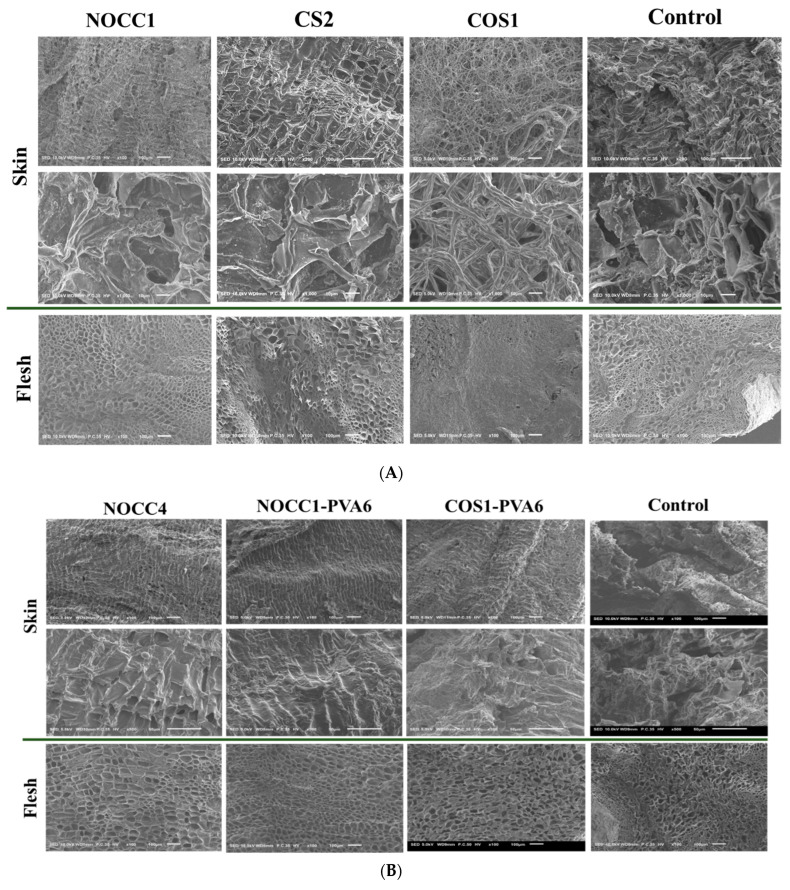

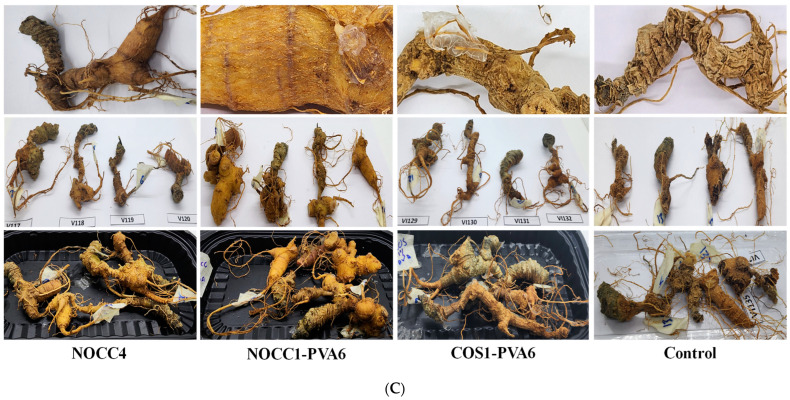
(**A**) SEM images of NL ginseng’s skin and flesh coated with the NOCC1, CS2, or COS1 solutions and non-coated (control) after a 35-day storage period, (*n* = 4). (**B**) SEM images of NL ginsengs’ skin and flesh coated with the NOCC4, NOCC1–PVA6, or COS1–PVA6 solutions and non-coated (control) after the 35-day storage period, (*n* = 4.). (**C**) Photograph images of NL ginsengs coated with the NOCC4, NOCC1–PVA1, or COS1–PVA6 solutions and non–coated (control) after the 35–day storage period, (*n* = 4).

Figure 5A displays the morphology of NL ginsengs coated with the chitosan-based solutions of NOCC1, COS1, CS2, and non–coated (control). The non-coated NL ginsengs were significantly dried and shriveled, with the appearance of numerous cracks on their skin and the significant collapse of some cellular structures in their flesh. Similarly, compared with the control group, NL ginsengs coated with the COS1 solution became completely dried, with their skin having a fibrous appearance, and their flesh becoming solid and shriveled. In contrast, those coated with NOCC1 or CS2 solutions maintained their texture better than the control group, with both their skin and flesh consisting of tightly arranged tissues, except for a slight collapse of some cellular structures in the flesh of NL ginseng coated with the CS2 solution.

In Figure 5B, SEM images of NL ginsengs coated with the film-forming solutions (NOCC4, NOCC1–PVA6, and COS1–PVA6) are shown to significantly improve their texture retention in both the flesh and skin tissues in comparison with the uncoated one. In particular, both NL ginsengs coated with the NOCC4 and NOCC1–PVA6 solutions retained their texture the best. However, those coated with the COS1–PVA6 solution exhibited a lightly dried and shrunken texture.

Photographs of the NL ginsengs coated with the film–forming solutions also showed notable differences compared with those non–coated, as shown in Figure 5C. Overall, all the coated NL ginseng samples were significantly fresher than the non-coated NL ones. The changes within the control group were evident, of which are the skin turning dark brown, drying, and shrinking flesh. As the best result, NL ginsengs coated with the NOCC1–PVA6 solution had the most vibrant appearance with bright yellow and smooth skin. Those coated with NOCC4 still appeared to have smooth skin, but their color changed to dark brown, and NL ginsengs coated with the COS1–PVA6 solution were faintly yellow and slightly dried.

#### 3.3.2. Skin Lightness and Weight Loss of NL Ginseng during Preservation

**Figure 6:**
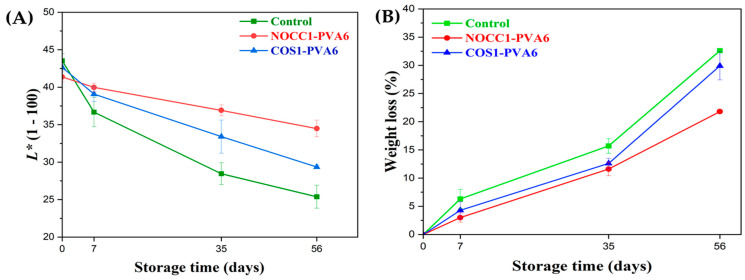
(**A**) Skin lightness and (**B**) weight loss of NL ginsengs coated with the blend solutions (NOCC1–PVA6 or COS1–PVA6) and non–coated (control) 0, 7, 35, and 56 days after storage. The data are presented as Mean ± SD (*n* = 4).

The skin lightness (L*) and weight loss values of NL ginsengs coated with the blend solutions (NOCC1–PVA6 or COS1–PVA6) and non-coated (control) during 56 days of storage are presented in Figure 6.

As shown in Figure 6A, the L* values of all NL ginsengs continuously decreased over the entire storage due to the progressive darkening of the ginseng skin color, resulting in a darker brown shade. In the beginning, there was no significant differences in the L* values of all NL ginseng samples, at around 42 (*p* > 0.05). In the first 7 days, the L* values of both the coated groups were around 39, significantly higher than the control at 36.6. Notably, on the 35th day, NL ginsengs coated with NOCC1–PVA6 experienced a slight decrease in the L* values to 36.9, which was statistically higher than the remaining figures coated with COS1–PVA6 and non–coated at 33.4 and 28.4, respectively. However, during the 56 days, there was a continuous decrease in the L* values of NL ginsengs coated with NOCC1–PVA6 to 34.4. Meanwhile, NL ginsengs coated with COS1–PVA6 and non–coated experienced a significant decline to 29.3 and 25.3, respectively.

Figure 6B shows that both coating formulations reduced the fresh weight loss of NL ginsengs during storage compared with the non-coated ones. In the first 7 days, both coated NL ginseng groups showed a slight reduction in the weight loss percentage, by approximately around 3–4.3% (*p* > 0.05), which was roughly half of that of the non–coated one at 6.3%. On the 35th day, the weight loss of NL ginsengs coated with NOCC1–PVA6 was 11.6%, which was significantly lower than that of the COS1–PVA6 and the control group at 12.6% and 15.7%, respectively. Notably, the fresh weight of all NL ginsengs exhibited a remarkable reduction after 56 days of storage. The NOCC1–PVA6 coated NL ginsengs demonstrated a weight loss of 21.8%, which was much lower than the other treatments (i.e., the COS1–PVA6 at 21.8% and the control at 32.6%).

#### 3.3.3. Sensory Evaluation of NL Ginseng during Preservation

**Figure 7:**
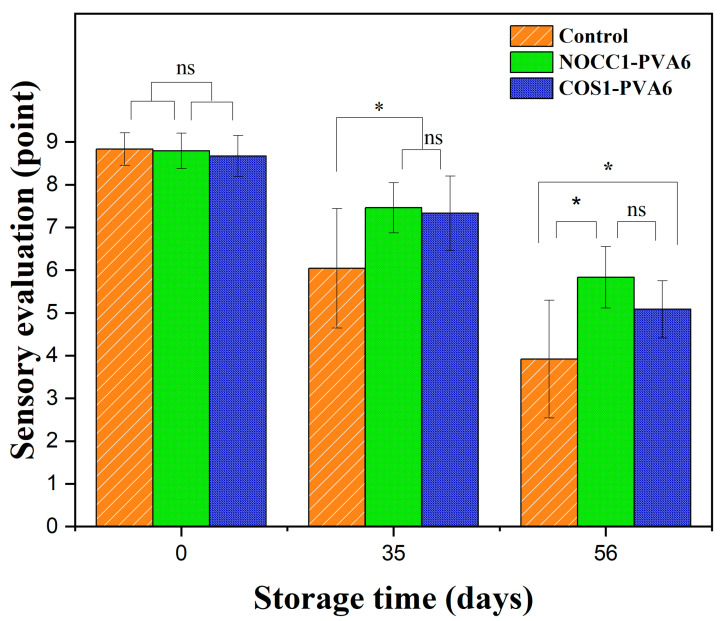
Sensory evaluation score of NL ginsengs coated with the blend solutions (NOCC1–PVA6 or COS1–PVA6) and non-coated (control) on 0, 35, and 56 days of storage. The data are presented as Mean ± SD (*n* = 4, *: *p* < 0.05, ns: *p* >0.05).

A sensory evaluation of NL ginsengs coated with the blend solutions (NOCC1–PVA6 or COS1–PVA6) and non-coated was conducted over 56 days of storage, and the corresponding data are shown in Figure 7. Overall, the sensory evaluation scores of all NL ginsengs significantly dropped over the observed periods (*p* < 0.05), with no statistically significant difference between the two coated groups (*p* > 0.05). Right after the coating process, all samples received comparable values of approximately 8.7 points (*p* > 0.05). After 35 days of storage, the sensory evaluation scores of the coated groups slightly declined to around 7.4 points, which were higher than that of the non-coated group at 6.0 points. However, on the 56th day, there was a significant reduction in the sensory evaluation values of NL ginsengs coated with NOCC1–PVA6 and COS1–PVA6, scoring 5.8 points and 5.08 points, respectively. These scores were notably higher than the control group’s score of 3.9 points (*p* < 0.05).

#### 3.3.4. Phytochemical Components of NL Ginseng during Preservation

**Figure 8:**
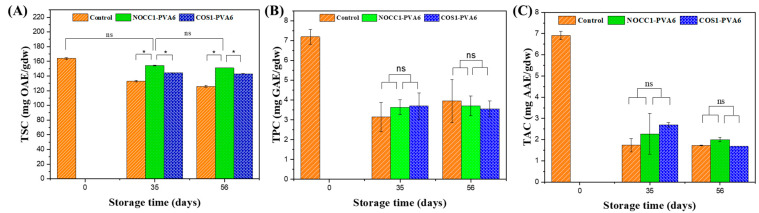
Total phytochemical content in NL ginsengs coated with the blend solutions (NOCC1–PVA6 or COS1–PVA6) and non-coated (control) in storage periods of 0 days, 35 days, and 56 days: (**A**) Total saponin content, (**B**) Total polyphenol content, (**C**) Total antioxidant capacity. The data are presented as Mean ± SD (*n* = 4, *: *p* < 0.05, ns: *p* >0.05).

The total phytochemical contents, including the saponin, polyphenol, and antioxidant capacity, in NL ginsengs coated with the blend solutions (NOCC1–PVA6 or COS1–PVA6) and non–coated 56 days after storage are presented in Figure 8.

Figure 8A shows that the TSC of all NL ginsengs continuously declined over the entire storage period, except for NL ginsengs coated with NOCC1–PVA6 on the 35th day, on which the TSC showed no significant change in the initial 35 days (*p* > 0.05). Before storage, i.e., at day 0, the TSC in NL ginsengs was determined at 164 mgOAE/gdw. On the 35th day, the TSC of NL ginsengs coated with NOCC1–PVA6 was 154 mgOAE/gdw, which was higher than those coated with COS1–PVA6 and non-coated, which were 144 mgOAE/gdw and 132 mgOAE/gdw, respectively. After 56 days of storage, there was a decrease in the TSC of NL ginsengs coated with NOCC1–PVA6 or COS1–PVA6 to approximately 152 mgOAE/gdw and 143 mgOAE/gdw, respectively, but both significantly higher than the non-coated control at 126 mgOAE/gdw.

The TPC and the TAC of all NL ginseng samples had a similar trend which are shown in Figure 8B,C. The values of these constituents significantly decreased after 35 days and remained stable until the 56th day. During the entire storage period, the TPC significantly decreased from 7.2 mgGAE/gdw to around 3.6 mgGAE/gdw. Similarly, the NL ginsengs experienced a reduction of their TAC from 6.9 mgAAE/gdw to around 22 mgAAE/gdw. There was no significant difference in both the TPC and the TAC between the coated and non–coated groups.

## 4. Discussion

As the most expensive and precious medicinal herb, NL ginseng urgently needs post-harvest preservation technology to prolong their shelf life as well as maintain their commercial and medicinal values. In this research, a preservation procedure of fresh NL ginseng was integrated into a multi-step to ensure maximum storage efficiency. In the initial processing, post-harvested NL ginsengs were immersed in sanitizer and rinsed with distilled water to discard soil, bacteria, mold, and fungi because the soil contains many of these pathogens and creates a favorable environment for them to penetrate and cause the rotting of ginseng. For example, the fungus *Fusarium solani*, found commonly in the soil and water, causes the decaying of ginseng, hence a decrease in its nutritional content [58]. According to previous research, a combination of sanitizer treatment and coating proved the preservation efficiency of ginseng [44]. Building on this knowledge, in our study, the coating method was applied to investigate the preservation of NL ginseng under optimal refrigerator conditions (~3 °C, ~40% RH).

We discovered the effects of the chitosan-based coating solutions on the NL ginseng preservation. Chitosan and its derivatives, including NOCC and COS, are biopolymers that can be commonly used for edible coating technology to preserve agricultural products [21,31,59]. Theoretically, the acidity of these coating solutions directly affects agricultural products’ quality and sensory values during exposed time. For example, American ginseng roots coated with a chitosan solution in 2% *v/v* of the organic acids (acetic, lactic, and levulinic) showed negative effects, whereas positive results were obtained with a reduced acid concentration of 1.5% *v/v* [44,60]. Therefore, it is essential to determine the acidity of coating solutions that has a specific effect on NL ginseng preservation. Our study also demonstrated the sensitivity of NL ginseng to the pH values of the coating solutions.

The acidity of the chitosan-based solutions is influenced by their chemical structures and the type of solvents used. [24]. In this research, the chemical functional groups of the CS solution were reported through FTIR, as shown in Figure 2A. From determining the presence of peaks in the results, the CS solution showed the amino groups which give it a positive charge under acidic conditions [21,61]. This positive charge makes chitosan soluble in acidic solutions below pH 6.0 due to the electrostatic repulsion between the same chitosan molecules. However, these amino groups are deprotonated in neutral or alkaline conditions, losing their positive charge, and becoming negatively charged. This charge leads to electrostatic attractions with other negatively charged species such as hydroxide ions (OH–) or anions. As a result, the CS solution forms an aggregation or gel, meaning it is insoluble in neutral or alkaline solutions. In our research, a chitosan solution (2% *w*/*v*) was found to be soluble with the lowest acetic acid level of pH 5.6.

In comparison with chitosan, the two derivatives of NOCC and COS exhibited enhanced solubility in neutral solvents due to modifications in their functional groups. The result showed that the COS solution had higher intensity peaks around 3300 cm^–1^, indicating increased amounts of the amino and hydroxyl groups. Additionally, there was a slight decrease in the wavenumber of the peak to 1630 cm^–1^, with a high intensity, suggesting an increase in the acetyl groups. This could be attributed to the deacetylation process of COS, resulting in variations such as the shorter chain length of amino acids and the increased amount of the hydrogen bonds [62]. Meanwhile, the NOCC solution appeared to have peaks of 1590 cm^−1^ and 1050 cm^–1^ with a higher intensity, indicating the presence of the carboxymethyl groups and the shorter polymer chains. These characteristics make NOCC and COS soluble in water at a neutral pH [26,63].

After the completion of the 35–day storage, the preservation results of NL ginsengs coated with the chitosan–based solutions (CS2, NOCC1, or COS1) at various pH values were reported and are shown in Figure 5A. The SEM images confirmed that NL ginsengs coated with the COS1 solution (pH 4.2) experienced detrimental effects, primarily due to complete dehydration when compared to the control group. In contrast, NL ginsengs coated with NOCC1 (pH 7.4) and CS2 (pH 5.6) exhibited superior preservation and retained their texture, thanks to effective water and freshness retention. This could be explained by the dehydration process changing the appearance of the NL ginsengs, affecting their dryness, freshness, and shape retention [64]. Furthermore, this finding aligns with previous studies that state that a coating solution of approximately pH 5.6 is reported to be optimal for fruit preservation [34]. Additionally, it is important to highlight that chitosan’s dissolution threshold is presented as pH 5.6. Therefore, any increase in pH beyond this point triggers precipitation, thus making it difficult to store this solution or mix it with another solution with a pH beyond 5.6. Therefore, the neutral pH solution (NOCC) is considered the best choice for preserving NL ginseng. However, these chitosan–based solutions limit its film–forming property due to low viscosity and their components, leading to the lack of a physical barrier as a protective mechanism.

The effect of the blend solutions on NL ginseng preservation is as follows.

In addition to the optimal cold temperature and high humidity of the storage conditions, employing appropriate packaging methods for agricultural products is also important to effectively preventing weight loss, protecting against physical damage, and reducing respiration rates [13,65]. One promising method that has drawn attention is the use of blend solutions, combining PVA with chitosan derivatives as the packaging and coating materials for preserving agriculture products effectively [37]. PVA is a synthetic polymer that has several advantages such as water solubility, biodegradability, adhesion, and especially customized and compatible properties with chitosan derivatives [38,66]. The utilization of a composite comprising both PVA and chitosan derivatives has shown significant improvements compared to using chitosan alone, as elaborated in the following discussion.

Several findings have proved that the addition of PVA to chitosan-based solutions improved the characteristics of the advanced coatings, such as their adhesive ability and film-forming, attributed to the interactions among their functional groups [67]. In this research, the FTIR results revealed the interaction between polymers in the blend solutions of NOCC–PVA and COS–PVA, as depicted in Figure 2B,C. Compared to their ingredients, the intensity of peaks in both the blend solutions at around 3250 cm^–1^ decreased, indicating the formation of hydrogen bonds between the hydroxyl groups of PVA and the carbonyl groups of NOCC/COS. Similar hydrogen bonding was observed between the PVA (1650 cm^−1^) and NOCC (1590 cm^−1^)/COS (1930 cm^−1^), causing a slight decrease in the peak wavenumber of NOCC–PVA (1570 cm^−1^) and COS–PVA (1600 cm^−1^). These findings align with previous studies exhibiting the formation of the hydrogen bonds [51,68]. Additionally, there are changes in other characteristics, including their viscosity and film-forming ability.

The characteristics of the blend films (NOCC1–PVA6 and COS1–PVA6) exhibited coating advantages compared to the single-component films, including their film-forming capability (Table 1), morphology and thickness (Figure 3), and mechanical properties (Figure 4 and Table 2). The results show that the viscosity, concentration, and ingredient of the coating solutions affected these characteristics. The NOCC4 solution (high viscosity of 1700 cP, 4% *w*/*v*) caused clumping on the surface of the coated substrate, whereas both solutions of NOCC1 and COS1 (low viscosity, 1% *w*/*v*) failed in film formation. Additionally, the morphology of the NOCC4 film exhibited the crystallization structure, with stress and elongation values at breaks measured at 4.46 MP and 0.874%, respectively. This result is consistent with other research, indicating that the NOCC4 film was brittle and had a lack of elasticity [55]. This was due to the nature of the biopolymer and the deacetylation process of NOCC synthesis, which disrupted the hydrogen bonds and shortened the polymer chains. On the other hand, the PVA6 solution (viscosity 25 cP, 6% *w*/*v*) formed a thin film with a semi–crystal structure [57]. This characteristic contributed to its great plastic deformation, as evidenced by the high elongation value at a strain of 20.51%. It was noted that the blend solutions (NOCC1–PVA6 and COS1–PVA6) achieved the ideal viscosity (240 and 185 cP, respectively) for facilitating the coating technique smoothly. Consequently, these blend films’ surfaces and cross-section morphology revealed both the crystal and semi-crystal structures, along with the formation of voids. This could be explained by the simultaneous presence of two immiscible phases of PVA and NOCC/COS, leading to the formation of empty spaces. This porous structure could enable ventilation, maintaining the appropriate respiration rate of coated NL ginseng [69]. Furthermore, the thickness of NOCC1–PVA6 and COS1–PVA6 were 26.07 and 34.9 µm, respectively, which were higher than those of PVA6 (12.8 µm). Notably, these thicknesses were comparable to those of commercial plastic bags (around 20 µm) [70]. These explanations were similar to the previous literature [34,65,70].

The final property of the blend films was the mechanical property. The stress–strain curves exhibited an improvement in the mechanical behavior of both blend films compared to the single–component films. It can be explained that the mechanical behavior of both the blend films was inherited from two types of natural and synthesized polymers [51]. As can be seen, there was a significant increase in the elongation at a strain of 6.84% (NOCC1–PVA6) and 11.72% (COS1–PVA6), related to the property of PVA. Similarly, the stress values at the breaks of NOCC1–PVA6 and COS1–PVA6 were 4.68 MPa and 4.38 MPa, respectively, owing to those of the chitosan derivatives. As expected, these formed films could be capable of protecting NL ginseng from external physical factors. The obtained results also agree with other reports working on PVA–chitosan blend films for food packaging thanks to barrier capability [53]. Furthermore, the preservation efficiency of NL ginsengs coated with these blend solutions was evaluated through changes in their appearance, quality durability, and sensory evaluation.

After the completion of the 35–day preservation, NL ginsengs coated with the film-forming solutions (NOCC4, NOCC1–PVA6, or COS1–PVA6) maintained their shelf-life better than the non-coated sample (Figure 5B,C). In more details, the non-coated NL ginsengs have a dried and shriveled texture, with their skin becoming cracked and having a pale-yellow color. These results, according to the literature [71], could be explained as a natural process of dehydration and senescence. The SEM results showed that both NL ginsengs coated with NOCC4 or NOCC1–PVA6 had a rigid cable texture and watery terrain. Moreover, the color of NL ginsengs coated with NOCC1–PVA6 were bright yellow, but those coated with NOCC4 turned dark brown. This can be explained by the fact that the coating layer plays an important role as a semi-permeable barrier, which controls the respiration rate. NL ginsengs coated with NOCC1–PVA6, a porous coating layer, can decrease their respiration rate and retain an aerobic metabolism of their tissue living at a low level. Meanwhile, those coated with NOCC4, due to a solid layer, could completely inhibit ventilation, thereby preventing the aerobic metabolism and triggering the anaerobic metabolism. In this process, the breakdown of glucose, releasing lactic acid or ethanol, causes physiologic deterioration like the color change of the ginseng skin [44]. Our findings agree with other research stating that the color of fresh ginseng roots coated and stored in a vacuum condition (without any ventilation) turned dark–brown after the storage period [13]. On the other hand, the appearance of NL ginseng coated with the COS1–PVA6 solution (pH 4.6) was slightly drier and paler than those coated with the NOCC1–PVA6 solution (pH 7.4). This result coincided with the following changes in their quality values, which were further observed up to 56 days.

Skin lightness, a reduction in fresh weight loss, and sensory quality were indicators to evaluate the efficiency of ginseng preservation as well as determine its market value [13]. Over a preservation period of 56 days, NL ginsengs coated with the blend solutions (NOCC1–PVA6 or COS1–PVA6) displayed a delayed color change (Figure 6A) and weight loss (Figure 6B) compared to the non-coated sample, leading to the substantial improvement of their sensory evaluation values (Figure 7). This can be explained by the fact that the coating, as a protective barrier, decreased the physiological metabolism and transpiration to prolong the ginsengs’ shelf life and freshness [14,72]. In addition, a standard coating solution should not induce remarkable change in the original sensory value of agricultural products [73]. Consequently, both blend solutions proved satisfactory, as both the L* values and the sensory evaluation values of the coated and non-coated NL ginsengs did not exhibit significant differences immediately after coating.

Simultaneously, the acidity of the COS1–PVA6 solution (pH 4.6) directly affected the coated NL ginsengs’ skin, making the decrease in the skin lightness and weight loss significantly more than that of those coated with the NOCC1–PVA6 solution (pH 7.4) during the storage period. However, the sensory evaluation of both the coated NL ginsengs was not statistically significantly different. Although the acidity of the COS1–PVA6 solution disrupted the metabolism in the coated NL ginsengs, its film-forming capability improved the sensory evaluation value during storage.

NL ginseng, like other ginseng species, are highly appreciated for their phytochemicals including saponins, polyphenols, and antioxidants [74,75]. Therefore, an effective preservation method should be capable of maintaining the ginseng’s phytochemical contents. Among these compounds, saponin in fresh NL ginseng has been known as a major biologically active component, with a high content indicative of its medicinal value [74]. Figure 8A shows both NL ginsengs coated with the blend solutions significantly retaining more TSC than the non-coated ones during the 56 days of storage. Moreover, TSC in NL ginsengs coated with NOCC1–PVA6 decreased less than in those coated with COS1–PVA6, with an especially negligible decrease in the first 35 days. This agrees with other research showing that the coating solution with a film-forming capability and a neutral pH plays a crucial role as a protective and balance barrier to reduce the metabolism rate of respiration, conserve energy, and minimize the loss of the phytochemical content, thereby delaying the loss of TSC in the coated NL ginseng during the preservation period under the cold storage conditions [14].

For TPC and TAC, the stability of the polyphenols and antioxidants in ginseng is relatively sensitive to environmental factors, such as temperature, pH, and light exposure [76]. In this study, Figure 8B,C show that TPC and TAC in all NL ginsengs considerably reduced by around 50% and 70%, respectively, for the initial 35 days and then remained unchanged for up to 56 days. There was no significant difference between the coated and non-coated groups during the observed periods. This suggests that the coating method did not have any impact on the preservation of TPC and TAC under the storage conditions applied in this research.

For postharvest agro-products, the determination of the optimal preservation duration to maintain their quality is one of utmost importance. The results indicate that the skin lightness, fresh weight, and sensory evaluation of NL ginsengs coated with the blend solutions decreased by 50% after 56 days of storage compared with those after 35 days of storage. Notably, TSC in NL ginsengs coated the NOCC1–PVA6 solution exhibited an insignificant decrease during the first 35 days and then continued to decrease afterwards. Consequently, we suggested that the most favorable duration for the preservation of NL ginseng is 35 days, and this is especially effective for NOCC1–PVA6.

## 5. Conclusions

This study highlights that the coating method by the blend solutions (NOCC1–PVA6 or COS1–PVA6) effectively preserved NL ginsengs for up to 56 days in refrigerator conditions (~3 °C; ~40% RH). Furthermore, NL ginseng coated with the NOCC1–PVA6 solution exhibited the best preservation efficiency in the first 35 days, thanks to the synergistic effects of a neutral pH value and film-forming capability. The results suggest that this coating solution has significant commercial potential for preserving NL ginseng. To further improve the preservation methods, future research is recommended to investigate and optimize bioactive agents loaded in the NOCC1–PVA6 formulation to prevent the degradation of the phytochemical component inside NL ginseng, as well as to extend the storage time.

## Figures and Tables

**Table 1:** Characteristics of the coating solutions: composition, concentration, pH, viscosity, and visual film–forming remark.

Sample	Concentration				pH	Viscosity (cP)	Remarks
	NOCC (*w*/*v*)	COS (*v*/*v*)	Chitosan (*w*/*v*)	PVA (*w*/*v*)			
NOCC4	4%	-	-	-	7.4	1700	Good film
NOCC1	1%	-	-	-	7.4	None	Phase separation
NOCC1–PVA6	1%	-	-	6%	7.4	240	Good film
COS1	-	1%	-	-	4.2	None	Non film
COS1–PVA6	1%	-	-	6%	4.6	185	Peeling problem
CS2	-	-	2%	-	5.6	None	Phase separation
PVA6	-	-	-	6%	6.5	25	Good film

**Table 2:** Tensile values of the NOCC4, NOCC1–PVA6, COS1–PVA6, and PVA6 films. The data are presented as Mean ± SD (*n* = 4).

Sample	Stress at Break (MPa)	Elongation at Break (%)
NOCC4	4.46 ± 0.137	0.87 ± 0.0347
NOCC1–PVA6	4.68 ± 0.2433	6.84 ± 0.378
COS1–PVA6	4.38 ± 0.1755	11.72 ± 0.574
PVA6	2.11 ± 0.046	20.51 ± 0.563

## Data Availability

The data will be provided by the first author or the corresponding author upon reasonable request, as it pertains to an ongoing study.
